# Transcriptomics of *In Vitro* Immune-Stimulated Hemocytes from the Manila Clam *Ruditapes philippinarum* Using High-Throughput Sequencing

**DOI:** 10.1371/journal.pone.0035009

**Published:** 2012-04-19

**Authors:** Rebeca Moreira, Pablo Balseiro, Josep V. Planas, Berta Fuste, Sergi Beltran, Beatriz Novoa, Antonio Figueras

**Affiliations:** 1 Instituto de Investigaciones Marinas, Consejo Superior de Investigaciones Científicas, Vigo, Spain; 2 Departament de Fisiologia i Immunologia, Facultat de Biologia, Universitat de Barcelona i Institut de Biomedicina de la Universitat de Barcelona, Barcelona, Spain; 3 Centros Científicos y Tecnológicos de la UB, Universitat de Barcelona, Barcelona, Spain; James Cook University, Australia

## Abstract

**Background:**

The Manila clam (*Ruditapes philippinarum*) is a worldwide cultured bivalve species with important commercial value. Diseases affecting this species can result in large economic losses. Because knowledge of the molecular mechanisms of the immune response in bivalves, especially clams, is scarce and fragmentary, we sequenced RNA from immune-stimulated *R. philippinarum* hemocytes by 454-pyrosequencing to identify genes involved in their immune defense against infectious diseases.

**Methodology and Principal Findings:**

High-throughput deep sequencing of *R. philippinarum* using 454 pyrosequencing technology yielded 974,976 high-quality reads with an average read length of 250 bp. The reads were assembled into 51,265 contigs and the 44.7% of the translated nucleotide sequences into protein were annotated successfully. The 35 most frequently found contigs included a large number of immune-related genes, and a more detailed analysis showed the presence of putative members of several immune pathways and processes like the apoptosis, the toll like signaling pathway and the complement cascade. We have found sequences from molecules never described in bivalves before, especially in the complement pathway where almost all the components are present.

**Conclusions:**

This study represents the first transcriptome analysis using 454-pyrosequencing conducted on *R. philippinarum* focused on its immune system. Our results will provide a rich source of data to discover and identify new genes, which will serve as a basis for microarray construction and the study of gene expression as well as for the identification of genetic markers. The discovery of new immune sequences was very productive and resulted in a large variety of contigs that may play a role in the defense mechanisms of *Ruditapes philippinarum*.

## Introduction

The Manila clam (*Ruditapes philippinarum*) is a cultured bivalve species with important commercial value in Europe and Asia, and its culture has expanded in recent years. Nevertheless, diseases produced by a wide range of microorganisms, from viruses to metazoan parasites, can result in large economical losses. Among clam diseases, the majority of pathologies are associated with the *Vibrio* and *Perkinsus* genera [1]–[3]. Although molluscs lack a specific immune system, the innate response involving circulating hemocytes and a large variety of molecular effectors seems to be an efficient defense method to respond to external aggressions by detecting the molecular signatures of infection [4]–[8]; however, not many immune pathways have been identified in these animals.

Although knowledge of bivalve immune-related genes has increased in the last few years, the available information is still scarce and fragmentary. Most of the data concern mussels and Eastern and Pacific oysters [9]–[14], and very limited information is available on the expressed immune genes of *R. philippinarum*. Recently, the expression of 13 immune-related genes of *Ruditapes philippinarum* and *Ruditapes decussatus* were characterized in response to a *Vibrio alginolyticus* challenge [15]. Also, a recent 454 pyrosequencing study was carried out by Milan *et al.* [16], who sequenced two normalized cDNA libraries representing a mixture of adult tissues and larvae from *R. philippinarum*. Even more recently Ghiselli et al. [17], have de novo assembled the *R. philippinarum* gonad transcriptome with the Illumina technology. Moreover, a few transcripts encoded by genes putatively involved in the clam immune response against *Perkinsus olseni* have been reported by cDNA library sequencing [18]. Currently (19/12/2011), there are 5,662 ESTs belonging to *R. philippinarum* in the GenBank database.

The European Marine Genomics Network has increased the number of ESTs for marine mollusc species particularly for ecologically and commercially important groups that are less studied, such as mussels and clams [19]. Unfortunately, most of the available resources are not annotated or well described, limiting the identification of important genes and genetic markers for future aquaculture applications. The use of 454-pyrosequencing is a fast and efficient approach for gene discovery and enrichment of transcriptomes in non-model organisms [20]. This relatively low-cost technology facilitates the rapid production of a large volume of data, which is its main advantage over conventional sequencing methods [21].

In the present work, we undertook an important effort to significantly increase the number of *R. philippinarum* ESTs in the public databases. Specially, the aim of this work was to discover new immune-related genes using pyrosequencing on the 454 GS FLX (Roche-454 Life Sciences) platform with the Titanium reagents. To achieve this goal, we sequenced the transcriptome of *R. philippinarum* hemocytes previously stimulated with different pathogen-associated molecular patterns (PAMPs) to obtain the greatest number of immune-related transcripts as possible. The raw data are accessible in the NCBI Short Read Archive (Accession number: SRA046855.1).

## Results and Discussion

### Sequence analysis and functional annotation

**Figure 1:**
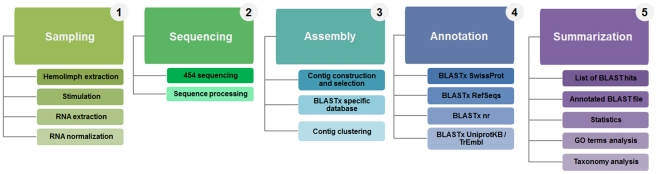
Flow chart summarizing work tasks and the data processing pipeline.

**Figure 2:**
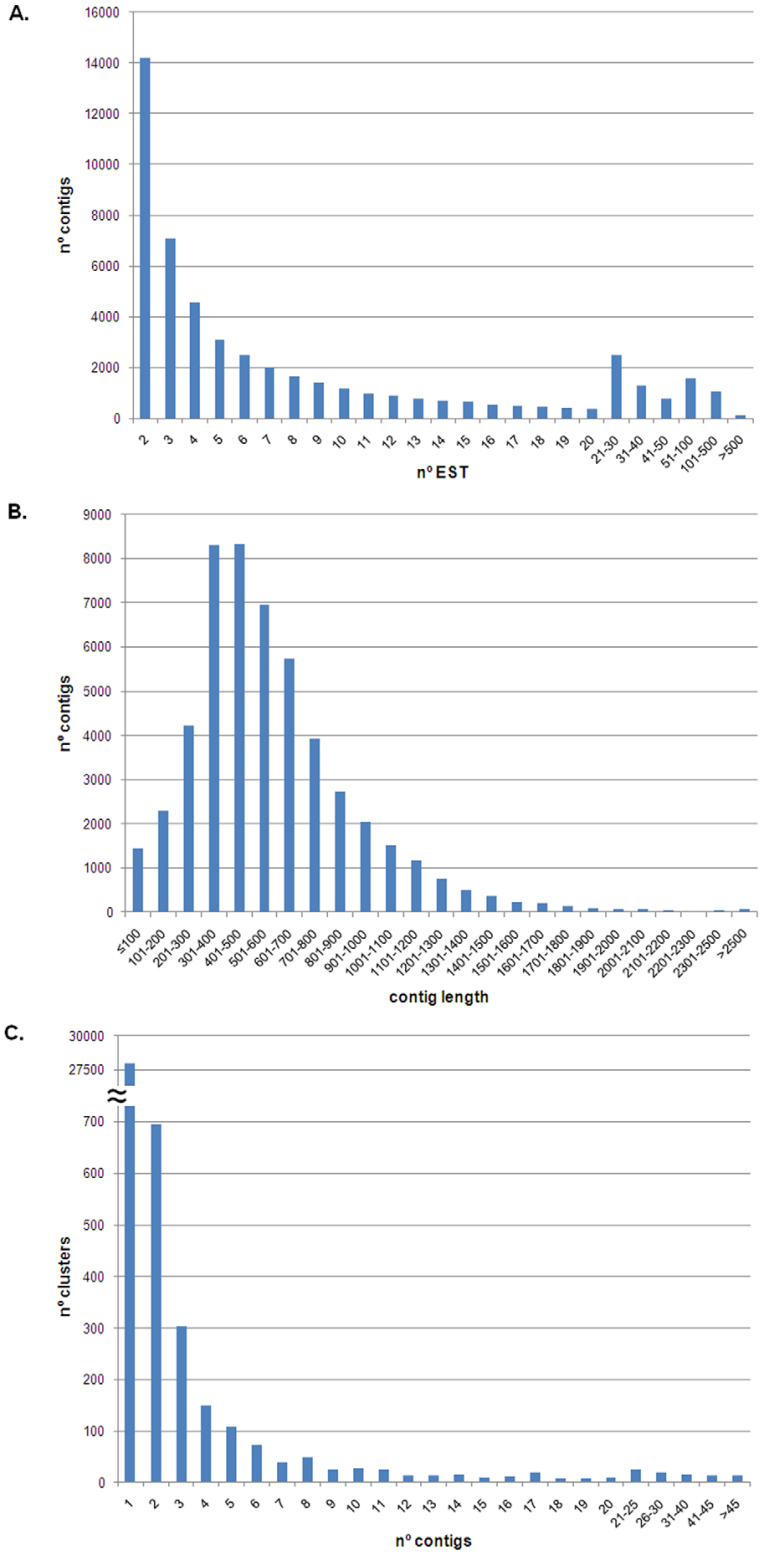
Transcriptome assembly statistics. **A:** Distribution of contig composition by EST. **B:** Distribution of contig length. **C:** Distribution of cluster composition by contigs.

The *R. philippinarum* normalized cDNA library was sequenced with 454 GS FLX technology as shown in Figure 1. Sequencing and assembly statistics are summarized in Table 1. Briefly, a total of 975,190 raw nucleotide reads averaging 284.1 bp in length were obtained. Of these, 974,976 exceeded our minimum quality standards and were used in the MIRA assembly. A total of 842,917 quality reads were assembled into 51,265 contigs, corresponding to 29.9 megabases (Mb). The length of the contigs varied from 40 to 5565 bp, with an average length of 582.4 bp and an average coverage of 5.7 reads. Singletons were discarded, resulting in 37,093 contigs formed by at least 2 ESTs, and 26,675 of these contigs were longer than 500 bp. Clustering the contigs resulted in 1,689 clusters with more than one contig. The distribution of contig length and the number of ESTs per contig, as well as the contig distribution by cluster are all shown in Figure 2.

**Table 1:** Summary of assembly and EST data.

**Sequences before filtering**	
Number of reads	975,190
Total Megabases	277.05
Average read length (bp)	284.1
N50 read length (bp)	356
**Sequences after filtering**	
Number of reads	974,976
Total Megabases	250.36
Average read length	256.78
N50 read length	338
**Assembly statistics**	
Number of reads assembled	842,957
Number of contigs	51,265
Total consensus Megabases	29.9
Average contig coverage	5.7
Average contig length	582.4
N50 contig length	677
Range contig length	40–5,565
Number of contigs >99 pb	49,847
Number of contigs >500 pb	26,675
Number of contigs with 2 reads	14,172
Number of contigs with >2 reads	37,093
Number of clusters	29,679
Number of clusters with 1 contig	27,990
Number of clusters with >1 contig	1,689
Percentage of contigs annotated	44.7
Percentage of annotated contigs by SwissProt	81.3
Percentage of annotated contigs by nr	16.2
Percentage of annotated contigs by RefSeq	2.5
Percentage of annotated contigs by UniprotKB/Trembl	0

Even though the knowledge of expressed genes in bivalves has increased in the last few years, it is still limited. Indeed, only 41,598 nucleotide sequences, 362,149 ESTs, 24,139 proteins and 704 genes from the class *Bivalvia* have been deposited in the GenBank public database (19/12/11), and the top entries are for the *Mytilus* and *Crassostrea* genera. For *Ruditapes philippinarum*, these numbers are reduced to 5,662 ESTs, 612 proteins and 12 genes. This evidences the lack of information which prompted the recent efforts to increase the number of annotated sequences of bivalves in the databases. For non-model species, functional and comparative genomics is possible after obtaining good EST databases. These studies seem to be the best resource for deciphering the putative function of novel genes, which would otherwise remain “unknown".

**Figure 3:**
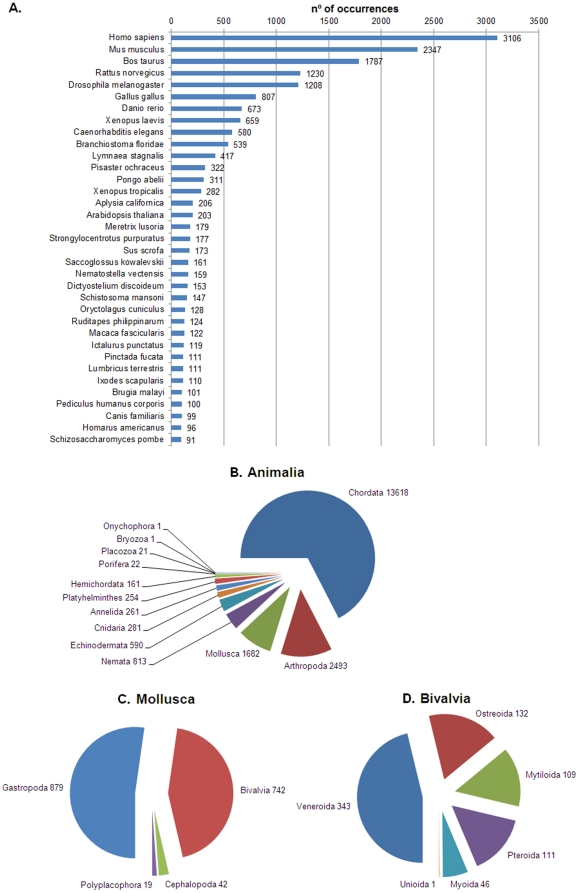
Taxonomic classification and distribution of annotated sequences. Numbers on bars and pie charts refers to the n° of occurrences. **A:** BLASTx results for the top 35 species matching sequences. **B:** Kingdom Animalia distribution. **C:** *Phylum* Mollusca distribution. **D:** Class Bivalvia distribution.

NCBI Swissprot, NCBI Metazoan Refseq, the NCBI non-redundant and the UniprotKB/Trembl protein databases were chosen to annotate the contigs that were at least 100 bp long (49,847). The percentage of contigs annotated with a cut off e-value of 10e-3 was 44.7%. Contig sequences and annotations are included in Table S1. Of these contigs, 3.26% matched sequences from bivalve species and the remaining matched to non-Bivalvia mollusc classes (4.13%), other animals (81.38%), plants (2.58%), fungi (1.78%), protozoa (1.50%), bacteria (4.95%), archaea (0.20%), viruses (0.21%) and undefined sequences (0.01%). As shown in Figure 3A, the species with the most sequence matches was *Homo sapiens* with 3,106 occurrences. The first mollusc in the top 35 list was *Lymnaea stagnalis* at position 11. The first bivalve, *Meretrix lusoria*, appeared at position 17. *R. philippinarum* was at position 25 with 124 occurrences. Notably, a high percentage of the sequences had homology with chordates, arthropods and gastropods (Figure 3B and C), and only 343 contigs matched with sequences from the *Veneroida* order (Figure 3D). These values can be explained by the higher representation of those groups in the databases as compared to bivalves and the quality of the annotation in the databases, which has been reported in another bivalve transcriptomic study [22]. The data shown highlight, once again, the necessity of enriching the databases with bivalve sequences.

**Figure 4:**
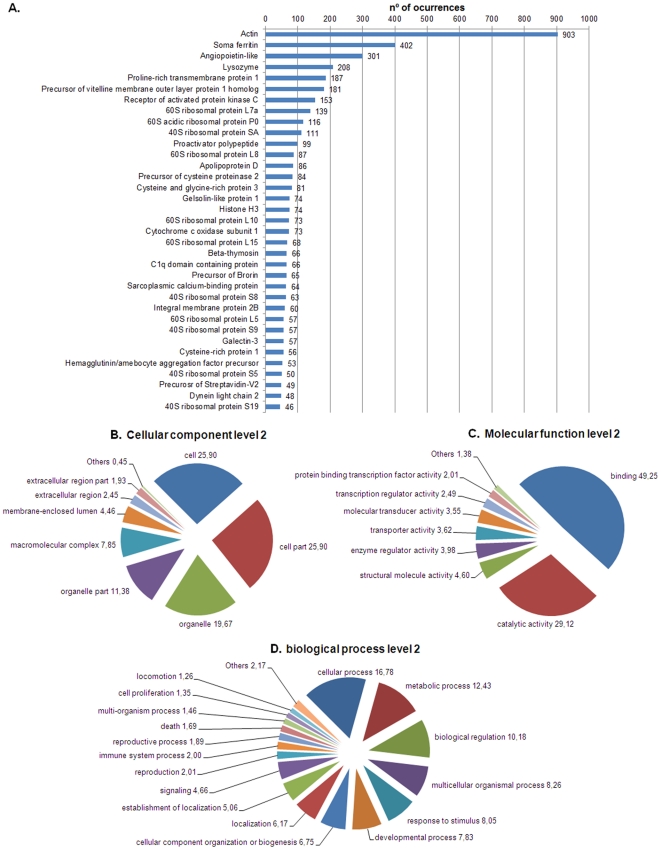
Classification of annotated sequences by BLASTx and GeneOntology Terms at level 2. **A:** The top 35 hit sequences by BLASTx. Numbers refer to the n° of occurrences **B:** Cellular component. **C:** Molecular function. **D:** Biological process. Numbers in B, C and D refer to the percentage of occurrences.

A detailed classification of predicted protein function is shown for the top 35 BLASTx hits (Figure 4A). The list is headed by actin with 903 occurrences, followed by ferritin, an angiopoietin-like protein and lysozyme. An abundance of proteins directly involved in the immune response was predicted for this 454 run; ferritin, lysozyme, C1q domain containing protein, galectin-3 and hemagglutinin/amebocyte aggregation factor precursor are immune-related proteins present on the top 35 list.

Ferritin has an important role in the immune response. It captures circulating iron to overcome an infection and also functions as a proinflammatory cytokine via the iron-independent nuclear factor kappa B (NF-κB) pathway [23]. Lysozyme is a key protein in the innate immune responses of invertebrates against Gram-negative bacterial infections and could also have antifungal properties. In addition, it provides nutrition through its digestive properties as it is a hydrolytic protein that can break the glycosidic union of the peptidoglycans of the bacteria cell wall [24]. The C1q domain containing proteins are a family of proteins that form part of the complement system. The C1q superfamily members have been found to be involved in pathogen recognition, inflammation, apoptosis, autoimmunity and cell differentiation. In fact, C1q can be produced in response to infection and it can promote cell survival through the NF-κB pathway [25]. Galectin-3 is a central regulator of acute and chronic inflammatory responses through its effects on cell activation, cell migration, and the regulation of apoptosis in immune cells [26]. The hemagglutinin/amebocyte aggregation factor is a single chain polypeptide involved in blood coagulation and adhesion processes such as self-nonself recognition, agglutination and aggregation processes. The hemagglutinin/amebocyte aggregation factor and lectins play important roles in defense, specifically in the recognition and destruction of invading microorganisms [27].

Other proteins that are not specifically related to the immune response but could play a role in defense mechanisms include the following: angiopoietin-like proteins, apolipoprotein D and the integral membrane protein 2B. In other animals, angiopoietin-like proteins (ANGPTL) potently regulate angiogenesis, but a subset also function in energy metabolism. Specifically, ANGPTL2, the most represented ANGPTL, promotes vascular inflammation rather than angiogenesis in skin and adipose tissues. Inflammation occurs via the a5b1 integrin/Rac1/NF-κB pathway, which is evidenced by an increase in leukocyte infiltration, blood vessel permeability and the expression of inflammatory cytokines (tumor necrosis factor-α, interleukin-6 and interleukin-1b) [28]. Apolipoprotein D (apoD) has been associated with inflammation. Pathological and stressful situations involving inflammation or growth arrest have the capacity to increase its expression. This effect seems to be triggered by LPS, interleukin-1, interleukin-6 and glucocorticoids and is likely mediated by the NF-κB pathway, as there are several conserved NF-κB binding sites in the apoD promoter (APRE-3 and AP-1 binding sites are also present). The highest affinity ligand for apoD is arachidonic acid, which apoD traps when it is released from the cellular membrane after inflammatory stimuli and, thus, prevents its subsequent conversion in pro-inflammatory eicosanoids. Within the cell, apoD could modulate signal transduction pathways and nuclear processes such as transcription activation, cell cycling and apoptosis. In summary, apoD induction is specific to ongoing cellular stress and could be part of the protective components of mild inflammation [29]–[31]. Finally, the short form of the integral membrane protein 2B (ITM2Bs) can induce apoptosis via a caspase-dependent mitochondrial pathway [32].

To avoid redundancy, the longest contig of each cluster was used for Gene Ontology terms assignment. A total of 23.05% of the representative clusters matched with at least one GO term. Concerning cellular components (Figure 4B), the highest percentage of GO terms were in the groups of cell and cell part with 25.9% in each; organelle and organelle part represented 19.67% and 11.38%, respectively. Within the molecular function classification (Figure 4C), the most represented group was binding with 49.25% of the terms, which was followed by catalytic activity (29.12%) and structural molecular activity (4.60%). With regard to biological process (Figure 4D), cellular and metabolic processes were the highest represented groups with 16.78% and 12.43% of the terms, respectively, which was followed by biological regulation (10.18%).

### Comparative analysis

Similarities between the *R. philippinarum* transcriptome and another four bivalve species sequences were analyzed by comparative genomics (*Crassostrea gigas* of the family Ostreidae, *Bathymodiolus azoricus* and *Mytilus galloprovincialis* of the family Mytilidae and *Laternula elliptica* of the family Laternulidae). This analysis could identify specific transcripts that are conserved in these five species. A Venn diagram was constructed using unique sequences from these databases according to the gene identifier (gi id number) of each sequence in its respective database: 207,764 from *C. gigas*, 76,055 from *B. azoricus*, 121,318 from *M. galloprovincialis* and 1,034,379 from *L. elliptica*. *C. gigas* was chosen because is the most represented bivalve species in the public databases. The other three species are bivalves that have been studied in transcriptomic assays.

**Figure 5:**
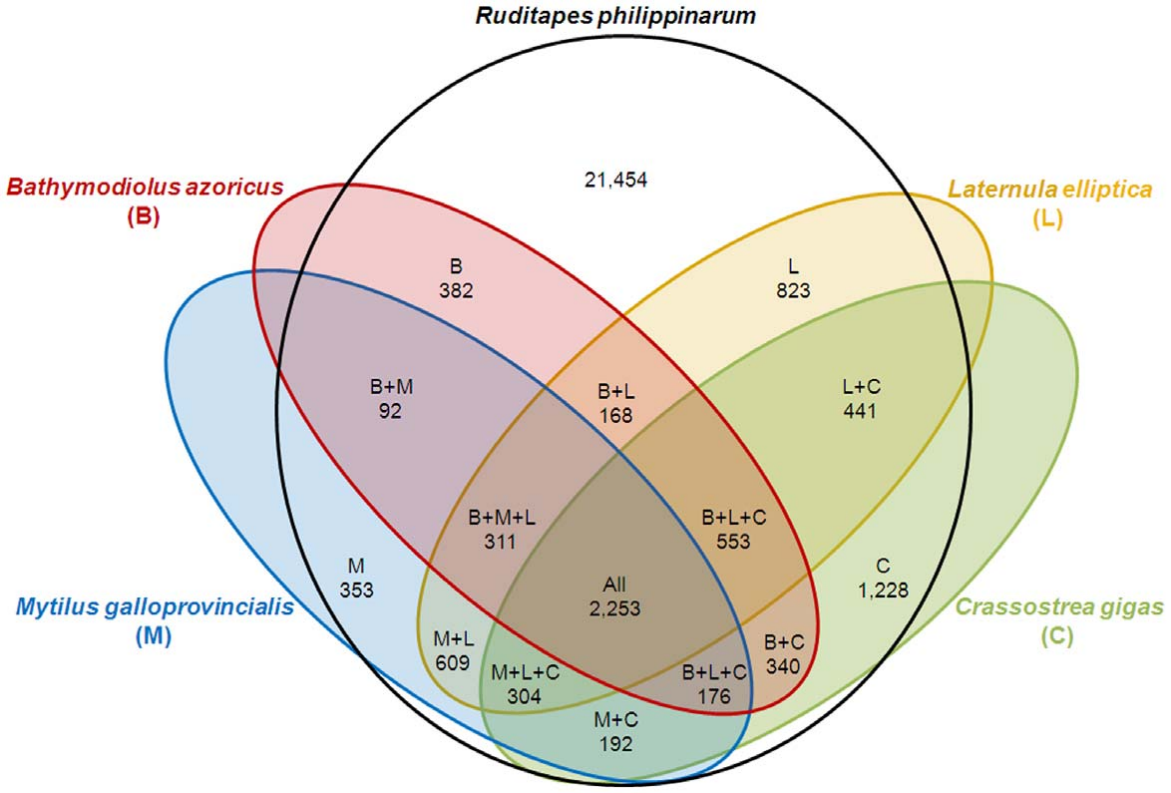
Venn diagram showing the comparison of *R. philippinarum* sequences with *B. azoricus* (B), *L.elliptica* (L), *M.galloprovincialis* (M) and *C.gigas* (C) known sequences. Numbers refer to the n° sequences belonging to each group.

Figure 5 shows that of the total 29,679 clusters, 72% were found exclusively in the *R. philippinarum* group, while only 7.59% shared significant similarity with all five species. The number of coincidences among other groups was very low (4.14% to 0.31% of sequences), suggesting that 21,454 new sequences were discovered within the bivalve group. The percentage of new sequences is very high compared to previous transcriptomic studies [33]–[34], in which the fraction of new transcripts was approximately 45%. One possible explanation for this discrepancy is the low number of nucleotide and EST sequences currently available in public databases for *R. philippinarum*, but these transcripts could also be regions in which homology is not reached, such as 5′ and 3′ untranslated regions or genes with a high mutation rate.

On the other hand, a comparison between our 454 results and the Milan *et al.* [16] transcriptome using a BLASTn approach is summarized in Table 2. It is worth noting that the number of hits for immune-related clusters is 1,120 (55.9%) when compared to Milan *et al.* transcriptome; consequently, there are 885 (44.1%) new clusters in our *R. philippinarum* immune transcriptome not represented in Milan *et al.* study. Moreover, 44% of our total results (13,059 clusters) were found in Milan *et al.* study; therefore, 56% of our total clusters (16,620) represent new information about Manila clam transcriptome compared to Milan *et al.* study.

**Table 2:** Comparison between Milan *et al.* and the current *R.philippinarum* transcriptome.

	Database (Milan et al. contigs)	
Query (current study)	% hits	% coverage
**TOTAL Clusters (29,679)**	44.0 (13,059)	66.6
**INMUNE-RELATED Clusters (2,005)**	55.9 (1,120)	67.6

### Immune-related sequences

**Figure 6:**
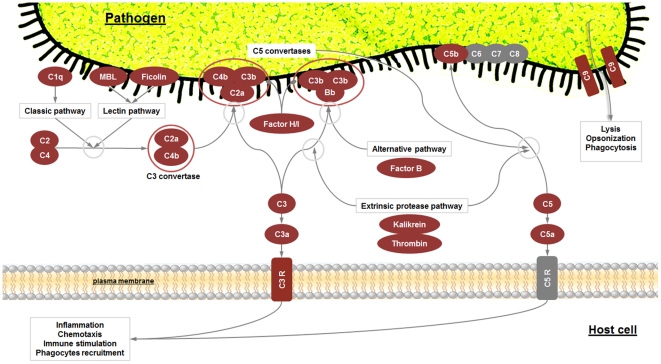
Complement pathway. Red boxes indicate proteins identified in our 454 results and grey boxes the absent ones. Connectors finishing in a circle indicate inhibition. C3R:C3 receptor; C5R: C5 receptor; MBL: Mannose-binding protein.

**Figure 7:**
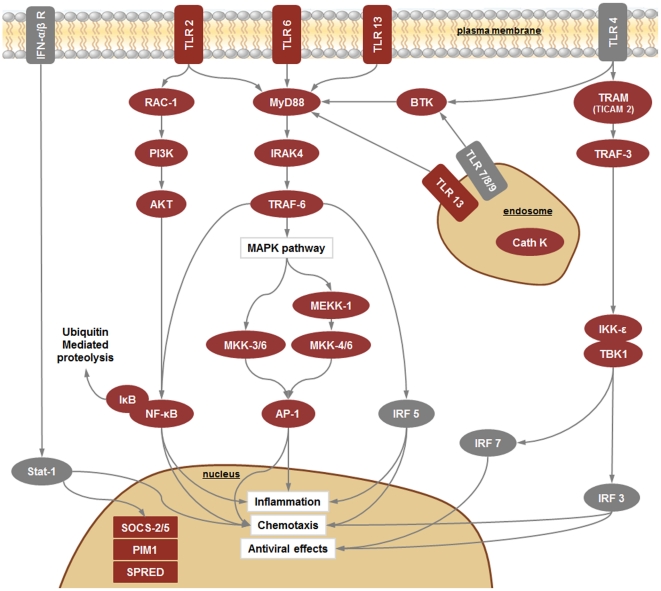
TLR signaling pathway. Red boxes indicate proteins identified in our 454 results and grey boxes the absent ones. Connectors finishing in a circle indicate inhibition. AKT: RAC-alpha serine/threonine-protein kinase = Protein kinase B; AP-1: Transcription factor AP-1 = Proto-oncogene c-Jun; BTK: Tyrosine-protein kinase BTK; Cath: Cathepsin; IFN-α/β R: Interferon alpha/beta receptor; IκB: Inhibitor of NF-κB; IKK-ε: Inhibitor of NF-κB kinase subunit epsilon; IRAK4: Interleukin-1 receptor-associated kinase 4; IRF: Interferon regulatory factor; MEKK: Mitogen-activated protein kinase kinase kinase; MKK: Mitogen-activated protein kinase kinase; MyD88: Myeloid differentiation primary response protein MyD88; NF-κB: Nuclear factor kappa B; PI3K: Phosphatidylinositol 3- kinase; PIM1: Proto-oncogene serine/threonine-protein kinase pim-1; Rac-1: Ras-related C3 botulinum toxin substrate 1; SOCS: Suppressor of cytokine signaling; SPRED: Sprouty-related, EVH1 domain-containing protein 2; Stat-1: signal transducer and activator of transcription 1; TBK1: TANK-binding kinase 1; TLR: Toll-like receptor; TRAF: TNF receptor-associated factor 3/6; TRAM: TIR domain-containing adapter molecule 2.

**Figure 8:**
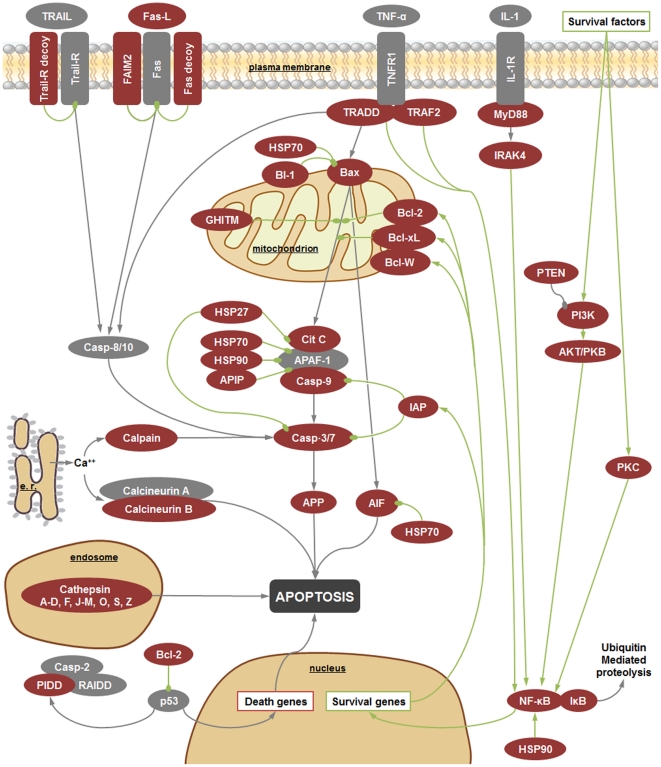
Apoptosis pathway. Red boxes indicate proteins identified in our 454 results and grey boxes the absent ones. Connectors finishing in a circle indicate inhibition. Green connectors highlight the survival pathways and grey ones indicate apoptosis. AIF: Apoptosis-inducing factor 1 mitochondrial; AKT/PKB: RAC-alpha serine/threonine-protein kinase = Protein kinase B; APAF-1: Apoptotic protease-activating factor 1; APIP: APAF1-interacting protein; APP: Amyloid beta A4 protein; Bax: Apoptosis regulator BAX; Bcl-2: Apoptosis regulator Bcl-2; Bcl-W: Bcl-2-like protein 2; Bcl-XL: Bcl-2-like protein 1; BI-1: Bax inhibitor 1; Casp: Caspase; Cit C: Cytochrome C; FAIM: Fas apoptotic inhibitory molecule 2; Fas: Apoptosis-mediating surface antigen FAS (CD95); Fas decoy: Decoy receptor for Fas ligand; Fas-L: Fas antigen ligand; GHITM: Growth hormone-inducible transmembrane protein = Transmembrane BAX inhibitor motif-containing protein 5; HSP: Heat shock protein; IAP: Inhibitor of apoptosis; IkB: Inhibitor of NF-kB; IL-1: Interleukin 1; IL-1 R: Interleukin 1 receptor; IRAK4: Interleukin-1 receptor-associated kinase 4; MyD88: Myeloid differentiation primary response protein MyD87; NF-kB: Nuclear factor kappa B; p53: Tumor suppressor p53; PI3K: Phosphatidylinositol 3- kinase; PIDD: p53-induced protein with a death domain; PKC: Protein kinase C; PTEN: Phosphatidylinositol-3,4,5-trisphosphate 3-phosphatase and dual-specificity protein phosphatase PTEN; RAIDD: Caspase and RIP adapter with death domain; TNF R1: Tumor necrosis factor receptor 1; TNF-a: Tumor necrosis factor alpha; TRADD: TNF receptor type 1-associated DEATH domain protein; TRAF2: TNF receptor-associated factor 2; TRAIL: TNF-related apoptosis-inducing ligand; TRAIL decoy: Decoy TRAIL receptor without death domain; TRAIL-R: TRAIL receptor.

*R. philippinarum* hemocytes were subjected to immune stimulation using several different PAMPs to enrich the EST collection with immune-related sequences. The objective was to obtain a more complete view of clam responses to pathogens. A keyword list and GO immune-related terms were used to find proteins putatively involved in the immune system. After this selection step, we found that more than 10% of the proteins predicted from the contig sequences had a possible immune function. Some sequences were found to be clustered in common, well-recognized immune pathways, such as the complement, apoptosis and toll-like receptors pathways, indicating conserved ancient mechanisms in bivalves (Figures 6, 7, 8).

### 1. Complement pathway

The complement system is composed of over 30 plasma proteins that collaborate to distinguish and eliminate pathogens. C3 is the central component in this system. In vertebrates, it is proteolytically activated by a C3 convertase through both the classic, lectin-induced and alternative routes [35]. Although the complement pathway has not been extensively described in bivalves, there is evidence that supports the presence of this defense mechanism. ESTs with homology to the C1q domain have been detected in the American oyster, *C. virginica* [36], the tropical clam *Codakia orbicularis* [37], the Zhikong scallop *Chlamys farreri* [38] and the mussel *M. galloprovincialis* [39]–[40]. More recently, a novel C1q adiponectin-like, a C3 and a factor B-like proteins have been identified in the carpet shell clam *R. decussatus* [41]–[42]. These data support the putative presence of the complement system in bivalves.

Our pyrosequencing results, using the BLASTx similarity approach, showed that the complement pathway in *R. philippinarum* was almost complete as compared to the KEGG reference pathway (Figure 6). Only the complement components C1r, C1s, C6, C7 and C8 were not detected.

### 2. Pattern recognition receptors (PRRs)

#### i. Lectins

Lectins are a family of carbohydrate-recognition proteins that play crucial self- and non-self-recognition roles in innate immunity and can be found in soluble or membrane-associated forms. They may initiate effector mechanisms against pathogens, such as agglutination, immobilization and complement-mediated opsonization and lysis [43].

Several types of lectins have been cloned or purified from the Manila clam, *R. philippinarum* [44]–[46], and their function and expression were also studied [18], [47]. Also, a Manila clam tandem-repeat galectin, which is induced upon infection with *Perkinsus olseni*, has been characterized [46].

Lectin sequences have been found in the stimulated hemocytes studied in our work: 23 of the contigs are homologous to C-type lectins (calcium-dependent carbohydrate-binding lectins that have characteristic carbohydrate-recognition domains), 115 are homologous to galectins (characterized by a conserved sequence motif in their carbohydrate recognition domain and a specific affinity for β-galactosides), 4 contigs have homology with ficolin A and B (a group of oligomeric lectins with subunits consisting of both collagen-like and fibrinogen-like domains) and 34 contigs have homology with other groups of lectins such as lactose-, mannose- or sialic acid-binding lectins.

#### ii. β-glucan recognition proteins

β-glucan recognition proteins are involved in the recognition of invading fungal organisms. They bind specifically to β-1,3-glucan stimulating short-term immune responses. Although these receptors have been partially sequenced in several bivalves, there is only one complete description of them in the scallop *Chlamys farreri* [48].

Two contigs with homology to the beta-1,3-glucan-binding protein were found in our study.

#### iii. Peptidoglycan recognition proteins

Peptidoglycan recognition proteins (PGRPs) specifically bind peptidoglycans, which is a major component of the bacterial cell wall. This family of proteins influences host-pathogen interactions through their pro- and anti-inflammatory properties that are independent of their hydrolytic and antibacterial activities. In bivalves, they were first identified in the scallops *C. farreri* and *A. irradians* [49], [50] and the Pacific oyster *C. gigas*, and from the latter four different types of PGRPs were identified [51].

Peptidoglycan-recognition proteins and a peptidoglycan-binding domain containing protein have been found for the first time in *R. philippinarum* in our results and were present 4 and 1 times, respectively.

#### iv. Toll-like receptors

Toll-like receptors (TLRs) are an ancient family of pattern recognition receptors that play key roles in detecting non-self substances and activating the immune system. The unique bivalve TLR was identified and characterized in the Zhikong Scallop, *C. farreri* [52].

TLR 2, 6 and 13 were present among the pyrosequencing results. TLR2 and TLR6 form a heterodimer, which senses and recognizes various components from bacteria, mycoplasma, fungi and viruses [53]. TLR13 is a novel and poorly characterized member of the Toll-like receptor family. Although the exact role of TLR13 is currently unknown, phylogenic analysis indicates that TLR13 is a member of the TLR11 subfamily [54] suggesting that it could recognize urinary pathogenic *E. coli* [55]. It has been demonstrated that TLR13 colocalizes and interacts with UNC93B1, a molecule located in the endoplasmic reticulum, which strongly suggests that TLR13 might be found inside cells and might play a role in recognizing viral infections [56]. Figure 7 summarizes the TLR signaling pathway with the corresponding molecules found in the *R. philippinarum* transcriptome.

### 3. Protease inhibitors

Pathogen proteases are important virulence factors that facilitate infection, diminish the activity of lysozymes and quench the agglutination capacity of hemocytes. Because protease inhibitors play important roles in invertebrate immunity by protecting hosts through the direct inactivation of pathogen proteases, many bivalves have developed protease inhibitors to regulate the activities of pathogen proteases [1]. Some genes encoding protease inhibitors were identified in *C. gigas* [57], *A. irradians* [58], *C. farreri* [59] and *C. virginica*; in the latter a novel family of serine protease inhibitors was also characterized [60]–[62].

A total of 23 contigs with homology to Serine, Cystein, Kunitz- and Kazal- type protease inhibitors and metalloprotease inhibitors were found among our results.

### 4. Lysozyme

Lysozyme was one of the most represented groups of immune genes in this transcriptome study with 208 contigs present. It is an antibacterial molecule present in numerous animals including bivalves. Although lysozyme activity was first reported in molluscs over 30 years ago, complete sequences were published only recently including those of *R. philippinarum* [24].

### 5. Antimicrobial peptides

Antimicrobial peptides (AMPs) are small, gene-encoded, cationic peptides that constitute important innate immune effectors from organisms spanning most of the phylogenetic spectrum. AMPs alter the permeability of the pathogen membrane and cause cellular lysis [63]. In bivalves, they were first purified from mussel hemocyte granules [64], [65]. In mussels, the AMP myticin C was found to have a high polymorphic variability as well as chemotactic and immunoregulatory roles [66], [67]. In clams, two AMPs with similarity to mussel myticin and mytilin [68] and a big defensin [69] are known.

We were able to detect 36 contigs with homology to different defensins: defensin-1 (American oyster defensin), defensin MGD-1 (Mediterranean mussel defensin) and the big defensin previously mentioned. Four contigs were similar to an unpublished defensin sequence from *Venerupis* ( = *Ruditapes*) *philippinarum*.

### 6. Heat shock proteins

The primary role of heat shock proteins (HSPs) is to function as molecular chaperones. Their up-regulation also represents an important mechanism in the stress response [70], and their activity is closely linked to the innate immune system. HSPs mediate the mitochondrial apoptosis pathway and affect the regulation of NF-κB [71]. HSPs are well studied in bivalves. For *R. philippinarum*, several assays have been developed to better understand the HSPs profile in response to heavy metals and pathogen stresses [72]–[74].

The most important and well-studied groups of HSPs were present in our *R. philippinarum* transcriptome (HSP27, HSP40/DnaJ, HSP70 and HSP90), but other, less common HSPs were also represented (HSP10, HSP22, HSP83 and some members from the HSP90 family).

### 7. Other immune molecules

Recently, several genes related to the inflammatory response against LPS stimulation have been detected in bivalves. Such is the case of the LPS-induced TNF-α factor (LITAF), which is a novel transcription factor that critically regulates the expression of TNF-α and various inflammatory cytokines in response to LPS stimulation. It has been described in three bivalve species: *Pinctada fucata* [75], *C. gigas* [76] and *C. farreri* [77].

Other TNF-related genes have been identified in the Zhikong scallop, such as a TNFR homologue [78] and a tumor necrosis factor receptor-associated factor 6 (TRAF6), which is a key signaling adaptor molecule common to the TNFR superfamily and to the IL-1R/TLR family [79]. Figure 7 shows that several components of the TLR signaling pathway that are present in our transcriptomic sequences (MyD88, IRAK4, TRAF-3 and -6, TRAM, BTK, RAC-1, PI3K, AKT, BTK and TANK).

### Pathogen sequences

**Figure 9:**
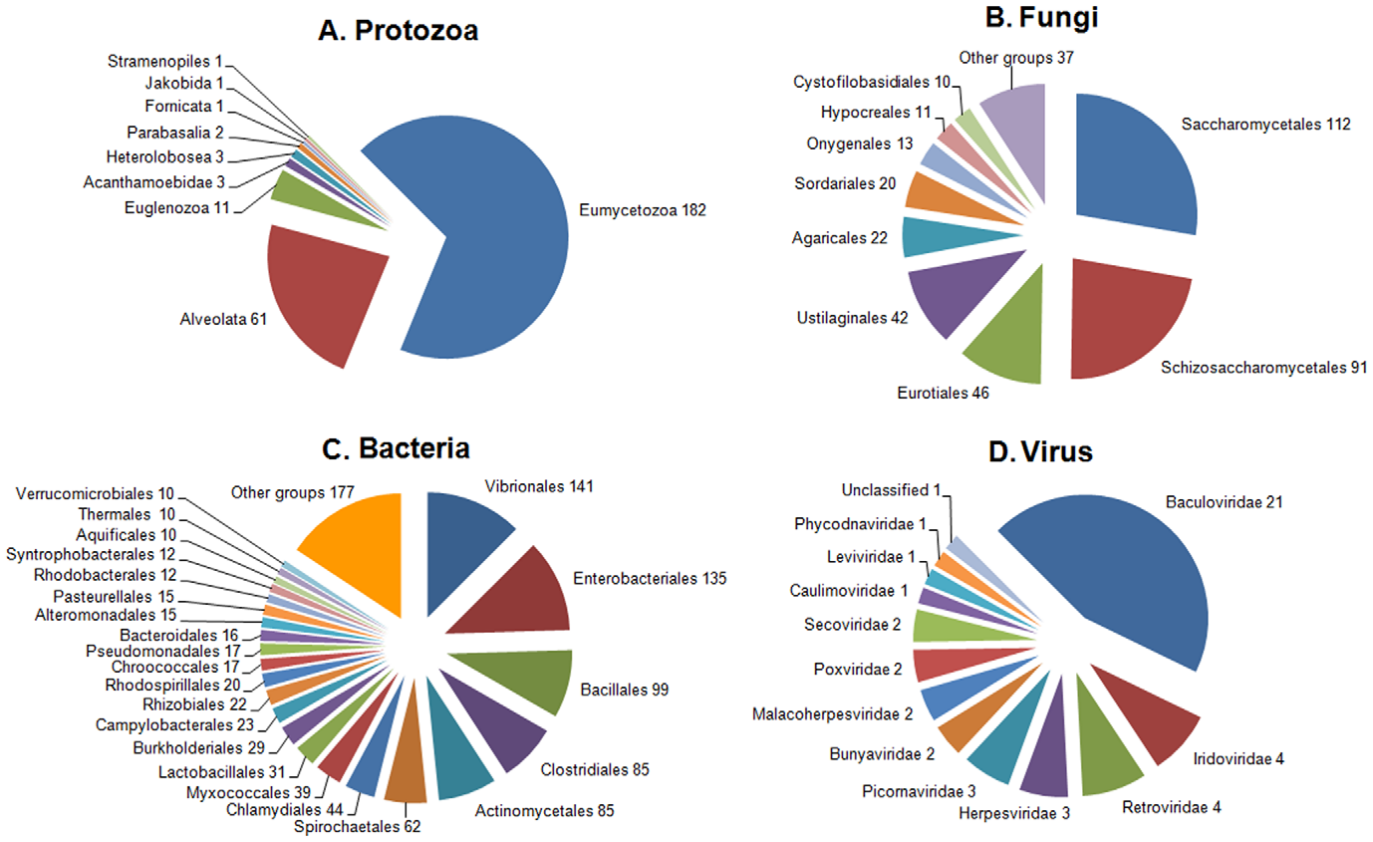
Classification of pathogen sequences. Numbers refer to the n° of occurrences. A: Protozoa. Results are observed at the phylum level. B: Fungi. Results are observed at the order level. C: Bacteria. Results are observed at the order level. D: Viruses. Results are observed at the family level.

A total of 1,918 contigs, 8.43% of those annotated, had homology with the main groups of putatively pathogenic organisms such as viruses (47 hits), bacteria (1,126 hits), protozoa (341 hits) and fungi (404 hits). Figure 9 displays the taxonomic classification of these sequences and Table 3 summarizes a list of the known bivalve pathogens found in our results.

**Table 3:** Putative bivalve pathogen sequences.

Pathogen species	n° contigs	Effects in bivalves
**Virus**		
*Ostreid herpesvirus 1*	2	Early stages mortality
**Bacteria**		
*Vibrio alginolyticus*	1	Bacilar necrosis
*Vibrio anguillarum*	2	Bacilar necrosis; Inhibited capacity for filtering
*Vibrio harveyi*	4	Adult and larval mortality
*Vibrio parahaemolyticus*	13	Bacilar necrosis
*Vibrio splendidus*	11	Bacilar necrosis; Summer mortality
Other *Vibrio* species	108	Bacilar necrosis
Genus *Pseudomonas*	16	Bacilar necrosis
Genus *Aeromonas*	2	Bacilar necrosis
Genus *Alteromonas*	1	Bacilar necrosis
Order *Chlamydiales*	42	Early stages mortality
Order *Ricketsiales*	6	Early stages mortality; Gill hyperplasia; Withering Syndrome
**Protozoa**		
*Perkinsus marinus*	2	Perkinsosis
**Fungi**		
*_*	401	Fungal disease

Bacteria constitute the main group found among the sequences not belonging to the clam. As filter-feeding animals, bivalves can concentrate a large amount of bacteria and it could be one of their sources of food [24]. Because *Vibrio* spp. are ubiquitous in aquatic ecosystems, it was expected that the *Vibrionales* order, with 141 hits, would be the most predominant. Several species of the *Vibrio* genus are among the main causes of disease in bivalves specifically causing bacillary necrosis in larval stages [80]. Is noticeable that sequences belonging to the causative agent of Brown Ring Disease in adults of Manila Clam, *Vibrio tapetis*, have not been found.

*Perkinsus marinus*, with 2 matches, is the only bivalve pathogen found within the protozoa (*Alveolata*) group. Perkinsosis is produced by species from the genus *Perkinsus*. Both *P. marinus* and *P. olseni* have been associated with mortalities in populations of various groups of molluscs around the world and are catalogued as notifiable pathogens by the OIE.

Viruses were the least represented among pathogens. The *Baculoviridae* family was the most predominant with 21 matches, but the corresponding sequences were inhibitors of apoptosis (IAPs) [81] that could also be part of the clam's transcriptome. Five viral families were found in our transcriptome study: *Iridoviridae*, *Herpesviridae*, *Malacoherpesviridae*, *Picornaviridae* and *Retroviridae*. A well-known bivalve pathogen was also identified, the ostreid herpesvirus 1, which has been previously been found to infect clams [82].

Fungi had 404 matches in our results. It is known that bivalves are sensitive to fungal diseases, which can degrade the shell or affect the larval bivalve stages [83], [84].

### Conclusions

This study represents the first *R. philippinarum* transcriptome analysis focused on its immune system using a 454-pyrosequencing approach and complements the recent pyrosequencing assay carried out by Milan *et al.* [16]. The discovery of new immune sequences was effective, resulting in an enormous variety of contigs corresponding to molecules that could play a role in the defense mechanisms. More than 10% of our results had relationship with immunity. This new resource is now gathered in the NCBI Short Read Archive with the accession number: SRA046855.1.

Our results will provide a rich source of data to discover and identify new genes, which will serve as a basis for microarray construction and gene expression studies as well as for the identification of genetic markers for various applications including the selection of families in the aquaculture sector. We have found sequences from molecules never described in bivalves before like C2, C4, C5, C9, AIF, Bax, AKT, TLR6 and TLR13, among others. As a part of this work, three immune pathways in *R. philippinarum* have been characterized, the apoptosis, the toll like signaling pathway and the complement cascade, which could help us to better understand the resistance mechanisms of this economically important aquaculture clam species.

## Materials and Methods

### Animal sampling and in vitro stimulation of hemocytes

*R. philippinarum* clams were obtained from a commercial shellfish farm (Vigo, Galicia, Spain). Clams were maintained in open circuit filtered sea water tanks at 15°C with aeration and were fed daily with *Phaeodactylum tricornutum* and *Isochrysis galbana*. Prior to the experiments, clams were acclimatized to aquaria conditions for one week.

A total of 100 clams were notched in the shell in the area adjacent to the anterior adductor muscle. A sample of 500 ul of hemolymph was withdrawn from the adductor muscle of each clam with an insulin syringe, pooled and then distributed in 6-well plates, 7 ml per well, in a total of 7 wells, one for each treatment. Hemocytes were allowed to settle to the base of the wells for 30 min at 15°C in the darkness. Then, the hemocytes were stimulated with 50 µg/ml of Polyinosinic∶polycytidylic acid (Poly I∶C), Peptidoglycans, ß- Glucan, *Vibrio anguillarum* DNA (CpG), Lipopolysaccharide (LPS), Lipoteichoic acid (LTA) or 1×10^6^ UFC/ml of heat-inactivated *Vibrio anguillarum* (one stimulus per well) for 3 h at 15°C. All stimuli were purchased from SIGMA.

### RNA isolation and cDNA production

#### Pyrosequencing

After stimulation, hemolymph was centrifuged at 1700 g at 4°C for 5 minutes, the pellet was resuspended in 1 ml of Trizol (Invitrogen) and RNA was extracted following the manufacturer's protocol. After RNA extraction, samples were treated with *Turbo DNase free* (Ambion) to eliminate DNA. Next, the concentration and purity of the RNA samples were measured using a *NanoDrop ND1000* spectrophotometer. The RNA quality was assessed in a Bioanalyzer 2010 (Agilent Technologies). From each sample, 1 µg of RNA was pooled and used for the production of normalized cDNA for 454 sequencing in the Unitat de Genòmica (SCT-UB, Barcelona, Spain).

Full-length-enriched double stranded cDNA was synthesized from 1,5 µg of pooled total RNA using MINT cDNA synthesis kit (Evrogen, Moscow, Russia) according to manufacturer's protocol, and was subsequently purified using the QIAquick PCR Purification Kit (Qiagen USA, Valencia, CA). The amplified cDNA was normalized using Trimmer kit (Evrogen, Moscow, Russia) to minimize differences in representation of transcripts. The method involves denaturation-reassociation of cDNA, followed by a digestion with a Duplex-Specific Nuclease (DSN) enzyme [85], [86]. The enzymatic degradation occurs primarily on the highly abundant cDNA fraction. The single-stranded cDNA fraction was then amplified twice by sequential PCR reactions according to the manufacturer's protocol. Normalized cDNA was purified using the QIAquick PCR Purification Kit (Qiagen USA, Valencia, CA).

To generate the 454 library, 500 ng of normalized cDNA were used. cDNA was fractionated into small, 300- to 800-basepair fragments and the specific A and B adaptors were ligated to both the 3′ and 5′ ends of the fragments. The A and B adaptors were used for purification, amplification, and sequencing steps. One sequencing run was performed on the GS-FLX using Titanium chemistry. 454 Sequencing is based on sequencing-by-synthesis, addition of one nucleotide, or more, complementary to the template strand results in a chemiluminescent signal recorded by the CCD camera within the instrument. The signal strength is proportional to the number of nucleotides incorporated in a single nucleotide flow. All reagents and protocols used were from Roche 454 Life Sciences, USA.

### Assembly and functional annotation

Pyrosequencing raw data, comprised of 975,190 reads, were processed with the Roche quality control pipeline using the default settings. *Seqclean* (http://compbio.dfci.harvard.edu/tgi/software/) software was used to screen for and remove normalization adaptor sequences, homopolymers and reads shorter than 40 bp prior to assembly. A total of 974,973 quality reads were subjected to MIRA, version 3.2.0 [87], to assemble the transcriptome. By default, MIRA takes into account only contigs with at least 2 reads. The other reads go into debris, which might include singletons, repeats, low complexity sequences and sequences shorter than 40 bp. NCBI *Blastclust* was used to group similar contigs into clusters (groups of transcripts from the same gene). Two sequences were grouped if at least 60% of the positions had at least 95% identity. The 51,265 contigs were grouped into a total of 29,679 clusters.

An iterative blast workflow was used to annotate the *R. philippinarum* contigs with at least 100 bp (49,847 contigs out of 51,265). Then, BLASTx [88] with a cut off value of 10e-3, was used to compare the *R. philippinarum* contigs with the NCBI Swissprot, the NCBI Metazoan Refseq, the NCBI nr and the UniprotKB/Trembl protein databases.

After annotation, Blast2GO software [89] was used to assign Gene Ontology terms [90] to the largest contig of a representative cluster (minimum of 100 bp). This strategy was used to avoid redundant results. Default values in Blast2GO were used to perform the analysis and ontology level 2 was selected to construct the level pie charts.

### Comparative analysis

To make a comparison between *R. philippinarum* and other bivalve species, the nucleotide sequences and ESTs from *C. gigas*, *M. galloprovincialis*, *L. elliptica and B. azoricus* were obtained from GenBank and from dedicated databases, when available. http://metagenomics.anl.gov/?page=DownloadMetagenome&metagenome=4442941.3, http://metagenomics.anl.gov/?page=DownloadMetagenome&metagenome=4442947.3, http://metagenomics.anl.gov/?page=DownloadMetagenome&metagenome=4442948.3, http://metagenomics.anl.gov/?page=DownloadMetagenome&metagenome=4442949.3 for *M. galloprovincialis* [91], http://www.ncbi.nlm.nih.gov/sra?term=SRA011054 for *L. elliptica* [92] and http://transcriptomics.biocant.pt:8080/deepSeaVent/?rvn=1 for *B. azoricus* [93]. Unique sequences from these databases (based on gi number) were used from each of the databases. These sequences were compared by BLASTn against the longest contig from each of 29,679 *R. philippinarum* clusters with a cut off e-value of 10e-05. Hits to *R. philippinarum* sequences were represented in a Venn diagram.

The comparison between our 454 results, the longest contig from each of 29,679 clusters, and the Milan *et al.* [16] transcriptome, contigs downloaded from RuphiBase (http://compgen.bio.unipd.it/ruphibase/query/), was made by BLASTn with a cut off e-value of 10e-05. Another analysis was carried out to compare just the longest contig from each of 2,005 clusters identified as immune-related and the Milan *et al.* contigs as well. The results were summarized in a table (Table 2). The percentage of coverage is the average % of query coverage by the best blast hit and the percentage of hits is the % of query with at least one hit in database, in parenthesis were added the total number of hits.

### Identification of immune-related genes

All the contig annotations were revised based on an immunity and inflammation-related keyword list (i.e. apoptosis, bactericidal, C3, lectin, SOCS…) developed in our laboratory to select the candidate sequences putatively involved in immune response. The presence or absence of these words in the BLASTx hit descriptions was checked to identify putative immune-related contigs. The remaining non-selected contigs were revised using the GO terms at level 2, 3 and 4 assigned to each sequence after the annotation step that had a direct relationship with immunity. Selected contigs were checked again to eliminate non-immune ones and distributed into functional categories.

Immune-related genes were grouped in three reference immune pathways (complement cascade, TLR signaling pathway and apoptosis) to describe each route indicated by our pyrosequencing results.

### Taxonomy analysis

To identify and classify the groups of organisms that had high similarity with our clam sequences, the Uniprot Taxonomy [94] was used except for the protozoa group. Because protozoa are a highly complex group, a specific taxonomy [95] was followed. Briefly, after the BLASTx annotation step all the hit descriptions included the species name (i.e. Homo sapiens) or a code (i.e. HUMAN) meaning that protein has been previously identified as belonging to that species. With such information sequences were classified in taxonomical groups and represented in pie charts.

## Supporting Information

Table S1 **List of contigs (e-value<10-3) of** ***Ruditapes philippinarum*** **including sequence, length, description (Hit description), accession number of description (Hit ACC), e-value obtained and database used for annotation (Blast).**
(XLSX)
